# Supplementary figures and images for: A positive feedback loop between sensory and octopaminergic neurons underlies nociceptive plasticity in Drosophila larvae
Source: PLoS Genet. 2026 Apr 28;22(4):e1012122. doi: 10.1371/journal.pgen.1012122 (PMC13152156; doi:10.1371/journal.pgen.1012122)

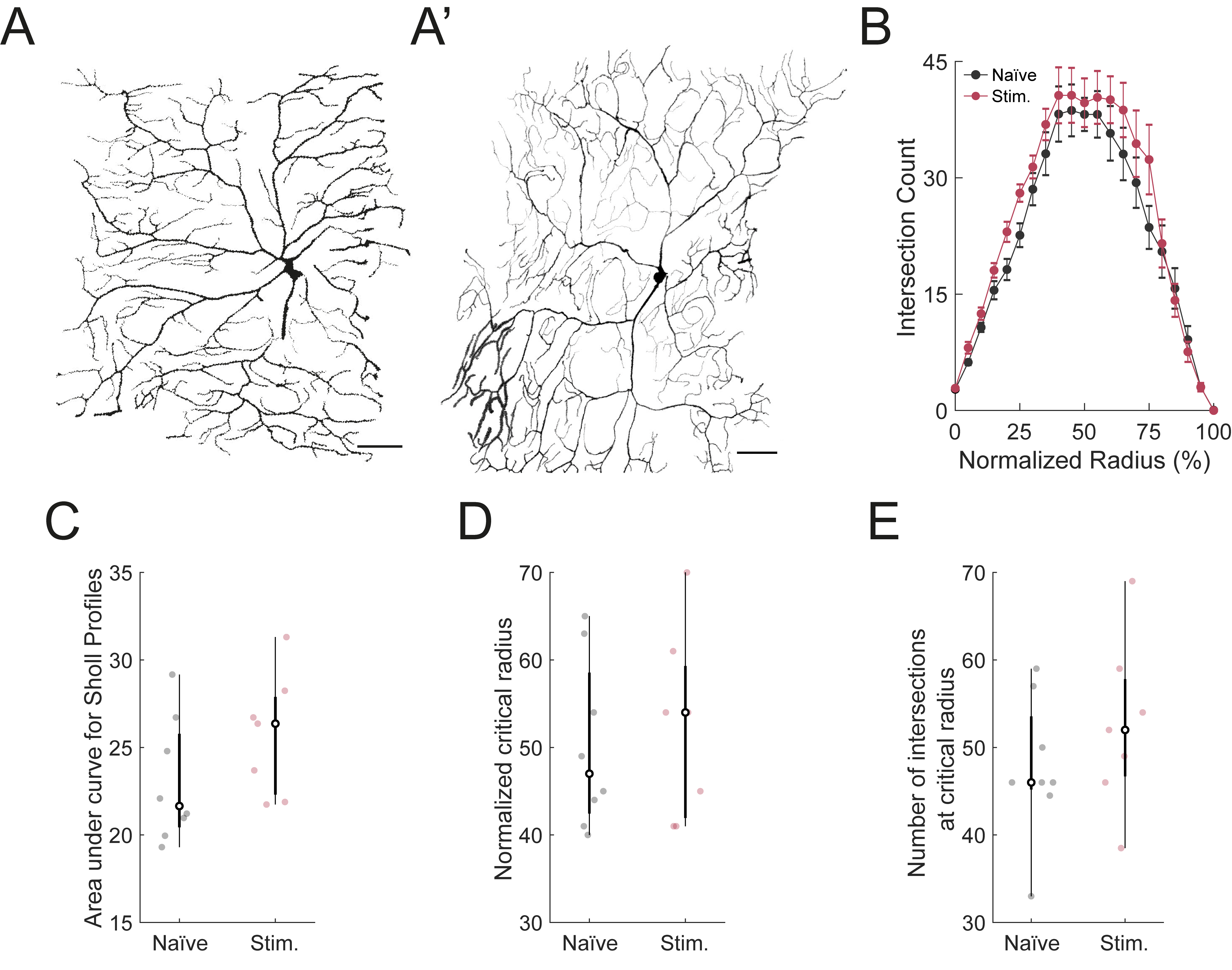

Supplement: S2 Fig — (A-A’) Representative dendritic arbour from naïve and developmentally stimulated larvae. Scale bar = 50 µm. (A) Naïve. (A’) Stimulated. (B) Average Sholl profiles for naïve (n = 7) and stimulated larvae (n = 8). Profiles were normalized to control for inter-individual variance in larval size. (C-E) Boxplots representing different core features of the normalized Sholl profiles in naïve (n = 7) and stimulated larvae (n = 8). (C) Area under the curve. (D) Critical radius. (E) Number of intersection at critical radius. (TIF) [file pgen.1012122.s002.tif]
