# Supplementary material for: A positive feedback loop between sensory and octopaminergic neurons underlies nociceptive plasticity in Drosophila larvae
Source: PLoS Genet. 2026 Apr 28;22(4):e1012122. doi: 10.1371/journal.pgen.1012122 (PMC13152156; doi:10.1371/journal.pgen.1012122)
Supplement: Attachment — Submitted filename: DraftResponseToReviewers_TO.pdf [file pgen.1012122.s013.pdf]

To the Editors of PLOS Genetics:

We have revised the manuscript, titled "**A positive feedback loop between sensory and octopaminergic neurons underlies nociceptive plasticity in *Drosophila* larvae**", authored by Boivin et al., and are grateful for the thoughtful suggestions and comments provided by the reviewers, which have greatly improved the clarity and rigor of our work. We have addressed all reviewer questions, concerns, and suggestions point by point. The main revisions include the following:

The main changes including the following.

1. We performed OAMB overexpression in C4da neurons experiment to test whether OAMB overexpression is sufficient to substitute for nociceptive activation during development (Figure Supplement 3G-I).
2. We conducted RT-qPCR experiments to confirm whether RNAi knockdown decreases the expression of OAMB or tbh as intended (Figure supplement 3D / Figure supplement 4C).
3. We performed OAMB RNAi knockdown during octopamine feeding (new Figure 5G-H) to confirm that effects of octopamine are mediated through OAMB in C4da neurons.
4. We carried out GRAB-OA imaging in C4da neurons during C4da neuron's activation (New Figure 6J-K).
5. We also added Figure 7 showing the hypothetical model.

Please find below our detailed, point-by-point responses to each reviewer comment. Our responses are provided in blue text. Line number refer to the revised manuscript without markup.

Reviewers' comments:

Comments to the Authors:
Please note here if the review is uploaded as an attachment.

Reviewer #1: The authors report on experiments using Drosophila melanogaster larvae, in which they stimulated nociceptive sensory cells either mechanosensorically or optogenetically. They find that after prolonged stimulation, depending on frequency and intensity, the animals' behavioral responses are either reduced or enhanced. They refer to these opposing processes as desensitization and sensitization, respectively, and attribute the sensitizing effect to the activity of specific octopaminergic (VUM) neurons. The study employs modern methodology (optogenetics, optical imaging) and several elegant technical approaches.

Overall, this is a well-conducted and informative study. The experimental design is mostly logical, sample sizes are appropriate, and statistical analyses are sound. The conclusions are clear and well supported by the data. I have only a few minor comments that the authors could address in a revision.

1. The terms desensitization and sensitization are typically defined very specifically. Desensitization usually refers to the reversal of a previously established sensitization. In the present case, the phenomenon described as "desensitization" would more appropriately be termed habituation. The authors should clarify this distinction and adjust the terminology accordingly throughout the manuscript.

We kindly thank the reviewer for this insightful comment. As we do not have evidence that high-frequency stimulation induces a state of sensitization prior to the observed decrease in nociceptive

responsiveness, we have replaced the term *desensitization* with *habituation* throughout the manuscript 99, 121, 189, 199, 296, 610, 671, 679 and 682.

2. The larvae display either habituation or sensitization depending on the duration, frequency, and intensity of stimulation. However, the main figures and text focus primarily on sensitization, with habituation data relegated to the supplementary materials. Since the switch between these behavioral states is particularly intriguing, I recommend presenting both phenomena in the main figures. It would also be informative to clarify how VUM neuron activity relates to habituation (or desensitization) under high-frequency stimulation. Do the octopaminergic neurons act as a switch mechanism between these behavioral outcomes?

We thank the reviewer for his feedback. We have moved our data that related to the shift between sensitization and habituation to the main figure where appropriate. In addition, we performed both imaging and behavioural experiments to assess whether VUM neurons contribute to the habituation phenomenon. Briefly, we found that VUM neurons exhibit a delayed response to noxious stimulations; however, their activity was not sufficient to induce habituation [Supplementary Figure 6; discussed between line 599 and 611]. These finding suggest that sensitization and habituation rely on distinct neural mechanisms, as noted in Discussion. We speculate that habituation induced by high frequency stimulation may instead involve serotonergic signaling, which has previously implicated in responses to continuous nociceptor simulation (Kaneko et. al 2017).

3. Figure 1B: The Y-axis label "larvae index" was confusing. Why is this expressed as an index rather than as the number of larvae? Clarifying this would help readers interpret the data correctly.

Thank you for pointing out this oversight. The label has been changed to "larval #" and the figure legend now clearly states that "Each row represents a single larva that was tracked continuously for the duration of the stimulation (line 148 and 149)".

4. Figure S3 shows an RNAi screen targeting various GPCRs. The data for the OAMB receptor, which was analyzed in more detail, are convincing. However, the RNAi efficiency should be directly demonstrated, at least for OAMB. For the other RNAi lines, please note explicitly that in cases where no behavioral effect was observed, it remains unclear whether the receptor is nonessential or whether the RNAi line was ineffective. Similarly, for the RNAi-mediated knockdown of tdc, molecular evidence (e.g., qPCR or immunostaining) should be provided to confirm that the gene product is effectively downregulated.

We thank the reviewer for pointing this flaw in our methodology. First, we added qPCR experiments to validate the efficiency of our RNAi-mediated knockdown of OAMB (Supplementary Figure 3D) and tbh (Supplementary Figure 4C) in pan-neuronal driver. In both cases, we observed a significant reduction in OAMB and tbh mRNA levels in larval brains. In addition, we have revised the text to acknowledge other receptors may contribute to the observed phenotype as we cannot exclude the possibility that incomplete knockdown efficiency (line302).

We note that RNAi lines used in this study (OAMB-RNAi-1:JF01732, OAMB-RNAi-2:JF01673, tbh-RNAi-1: JF02746, tbh-RNAi-2: HMS05829) have been previously validated and employed to knock down OAMB and tbh in prior studies. Please see below several examples of publications.

JF01732:

Deady, L. D., & Sun, J. (2015). A follicle rupture assay reveals an essential role for follicular adrenergic signaling in Drosophila ovulation. PLoS Genetics, 11(10), e1005604.

Bakshinska, D., Liu, W. Y., Schultz, R., Stowers, R. S., Hoagland, A., Cypranowska, C., ... & Isacoff, E. Y. (2025). Synapse-specific catecholaminergic modulation of neuronal glutamate release. Proceedings of the National Academy of Sciences, 122(1), e2420496121.

JF01673:

El-Kholy, S. E., Afifi, B., El-Husseiny, I., & Seif, A. (2022). Octopamine signaling via OctαR is essential for a well-orchestrated climbing performance of adult Drosophila melanogaster. Scientific Reports, 12(1), 14024.

Blaum, N., Ghelani, T., Götz, T. W., Chronister, K. S., Bengochea, M., Ceresnova, L., ... & Walter, A. M. (2025). Monoamine-induced diacylglycerol signaling rapidly accumulates Unc13 in nanoclusters for fast presynaptic potentiation. Proceedings of the National Academy of Sciences, 122(34), e2514151122.

JF01732 & JF01673:

Wang, Q. P., Lin, Y. Q., Zhang, L., Wilson, Y. A., Oyston, L. J., Cotterell, J., ... & Neely, G. G. (2016). Sucralose promotes food intake through NPY and a neuronal fasting response. Cell metabolism, 24(1), 75-90.

Imura, E., Shimada-Niwa, Y., Nishimura, T., Hückesfeld, S., Schlegel, P., Ohhara, Y., ... & Niwa, R. (2020). The Corazonin-PTTH neuronal axis controls systemic body growth by regulating basal ecdysteroid biosynthesis in Drosophila melanogaster. Current Biology, 30(11), 2156-2165.

Yoshinari, Y., Ameku, T., Kondo, S., Tanimoto, H., Kuraishi, T., Shimada-Niwa, Y., & Niwa, R. (2020). Neuronal octopamine signaling regulates mating-induced germline stem cell increase in female Drosophila melanogaster. Elife, 9, e57101.

JF02746:

Castellanos, M. C., Tang, J. C., & Allan, D. W. (2013). Female-biased dimorphism underlies a female-specific role for post-embryonic Ilp7 neurons in Drosophila fertility. Development, 140(18), 3915-3926.

Sun, M., Ma, M., Deng, B., Li, N., Peng, Q., & Pan, Y. (2023). A neural pathway underlying hunger modulation of sexual receptivity in Drosophila females. Cell Reports, 42(10).

Yu, Y., Huang, R., Ye, J., Zhang, V., Wu, C., Cheng, G., ... & Wang, L. (2016). Regulation of starvation-induced hyperactivity by insulin and glucagon signaling in adult Drosophila. Elife, 5, e15693.

HMS05829:

Yoshinari, Y., Ameku, T., Kondo, S., Tanimoto, H., Kuraishi, T., Shimada-Niwa, Y., & Niwa, R. (2020). Neuronal octopamine signaling regulates mating-induced germline stem cell increase in female Drosophila melanogaster. Elife, 9, e57101.

Martin-Diaz, J., & Herrera, S. C. (2024). A stem cell activation state coupling spermatogenesis with social interactions in Drosophila males. Cell Reports, 43(8).

5. There is a pressing need to improve the presentation and use of genetic controls for experiments involving RNAi lines. In some cases (e.g., Figure 3), only a "no RNAi" group is indicated as the contro (specified only in the supplementary table). However, it is standard practice—and essential for scientific rigor—to include both parental control genotypes in the analysis: the UAS-RNAi line crossed to a control background (without the driver), and the Gal4 driver line crossed to a control background (without the RNAi construct). Both heterozygous parental controls must be tested alongside the Gal4>UAS:RNAi experimental cross to rule out background or driver-related effects. Including these controls is not optional but required to meet current methodological standards in Drosophila genetics.

We thank the reviewer for raising this concern. In most experiments, we used the UAS>GAL4 system to drive expression of both CsChrimson (opsin) and RNAi in C4da neurons, which is essential for activating nociceptive neurons (C4da) during development. For this reason, a no-driver control would preclude optogenetic activation and therefore prevent us from examining the phenomenon under study.

To address the possibility that UAS-RNAi alone affects behavior, we included UAS-RNAi only controls in octopamine-feeding experiments with OAMB-RNAi knockdown (new Figure 5F). These data show that the parental background does not significantly affect behaviour in this chemical assay, supporting the conclusion that the observed effects are specific to the experimental manipulation.

In summary, this is a strong and elegant study that provides valuable insight into the mechanisms of nociceptive plasticity in Drosophila larvae. With some clarifications and several additions, the manuscript will be even clearer and more comprehensive. I congratulate the authors on this successful and informative work.

Reviewer #2: *The manuscript titled 'A positive feedback loop between sensory and octopaminergic neurons underlies nociceptive plasticity in Drosophila larvae' by Boivin et al. investigates how developmental activation of nociceptive C4da neurons in Drosophila larvae drives experience-dependent sensitization of nociceptive behavior. The work makes three major advances: (1) It establishes that sensitization arises without morphological remodeling of C4da dendrites but instead through enhanced neuronal activity, pointing to functional rather than structural mechanisms of adaptation. (2) It identifies the octopamine and its receptor OAMB as a critical modulator of sensitization. (3) It suggests direct connectivity between C4da neurons and the VUM octopaminergic motor neurons (such direct sensory neuron-to-motor neuron connections are thought to be rare), and demonstrates that VUM activation is sufficient to induce sensitization of behavior. Together, these findings highlight a neuromodulator-driven mechanism of plasticity that tunes nociceptive sensitivity and behavioral output according to developmental experience, paralleling vertebrate central sensitization and pointing to an evolutionarily conserved strategy in which adrenergic/octopaminergic signaling dynamically calibrates nociceptive circuits.*

The manuscript, including experiments, are well considered and we request only minor revisions. These suggestions are listed below in the order of the text.

1. *Line 110: "optogenetic" should be revised to "optogenetically".*
   Line 111: "specially" should be revised to "especially".
   Lines 115-116: ", with the" should be revised to "occuring".
   Line 118: "spend" should be revised to "spent"

We appreciate the reviewer for pointing out the typos and errors. We have revised the text as requested.

2. Figure 1B: please label the conditions.

We thank the reviewer for the suggestion. Accordingly, we have added the condition labels (Stim. or Naïve) to Figure 1B.

3. Figure 3D: please expand the figure caption to explain all the symbols. Alternatively, simplify the schematic to show each sensory neuron using the same symbol and highlight just the C4da neurons in red.

We thank the reviewer for this suggestion. We have revised the figure legend as following.

Figure 3D: Schematic of the larval somatosensory system. Triangles and circles represent external sensory neurons, rectangles represent the ciliated chordotonal neurons, and rotated square represent multidendritic neurons. The C4da neurons (vdaB, v'ada, ddaC) are highlighted in red (line 330 – 332).

4. Figure 3E: calcium imaging is shown only for ddaC but the caption states it is shown for all C4da neurons.

We thank the reviewer for comments. We have revised the figure caption to "Calcium imaging of ddaC neuronal responses" instead of "C4da (line 333).

5. Mentioning earlier in the text that C4da neurons is a class of sensory neurons that comprises three neurons may improve readability when the manuscript switches from considering C4da neurons as a whole versus individual neurons.

We thank the reviewer for this comment. To improve clarity, we now explicitly state earlier in the manuscript that C4da class neurons comprises three individual sensory neurons, consistent with our description of C4da neurons elsewhere (line 344-346).

6. Figure 3G: consider adding a non C4da neuron here as a negative control.

Thank you for reviewers' suggestions. We revised figure 3G to include non-C4da neurons as negative control.

7. Figure 4A: why do the UAS-tbh-RNAi(1) and UAS-tbh-RNAi(2) control lines differ significantly in their rolling probability? Please explain.

We thank the reviewer for pointing out this oversight. The significant difference between the UAS-tbh-RNAi(1) and UAS-tbh-RNAi(2) control lines likely reflects background effects, as different RNAi lines can exhibit distinct behavioral baselines (probability of rolling). Despite these baseline differences, we consistently observed that developmental stimulation induced sensitization. Importantly, tbh RNAi knockdown partially but significantly reduced this sensitization across lines.

8. Figure 4C: please plot UAS-GtACR1 controls as well.

We thank the reviewer for the suggestions and have added another control (only tdc2-GAL4 control which is missing in first submission) in Figure 4C.

9. Figure 6A: explain what "nc82" stains for in the figure caption. Add contextual details about the view and location of the slices that have been shown.

We thank the reviewer for the suggestions and have revised the figure legend as follows (line 540-546). Figure 6 (A-A') *Anatomical relationship between tdc2+ neurons and C4da neurons at abdominal segment A4. Tdc2-LexA was used to drive expression of mCD8::GFP (green) in tdc2+ neurons, while ppk1.9-GAL4 drove mCD8::RFP (magenta), tagging the cell membrane of both neuron population. nc82 (blue) is used as a counterstaining for the neuropil and indicates active zones within the brain. (A) Dorsal view. (A') Transverse view. Both views: Arrow heads and triangle indicate points of contact between the abdominal VUM arbor and the C4da axon terminal and point at the same coordinates. Dashed lines indicate the position at which views intersect. Scale bar = 20 µm.*

10. Add a supplementary figure showing the absence of co-staining between tdc2+ neurons and the other octopaniergic neurons (i.e., DUMs, ABLKs, and VPMs).

We kindly thank the reviewer's suggestion. We added new supplemental figures (Supplemental Figure 5) to show all tdc2+ neurons. New supplemental figures show that all tdc2+ neurons including DUMs and VPMs.

11. Consider confirming the synaptic connections between the VUMs and C4da neurons in the larval Drosophila melanogaster VNC connectome and including these data as a figure panel.

We thank the reviewers for their suggestions. We examined the connectome data (Ohyama et al., 2015); however, VUM neurons are reported to be immature in first-instar larvae and therefore were not reconstructed in the connectome dataset (Zarin et al., 2019). We have added this information to the Results section (line 523-524).

12. We recommend adding a summary schematic of the proposed circuit to Figure 6.

We thank the reviewer for the suggestion. We agree that a separate schematic would help clarify the model. Therefore, we have created a standalone schematic, now presented as Figure 7.

13. Figures S2 should be presented before S3 in the supplementary document.

We thank the reviewers for pointing this out. We have changed the order of Figure S2 and S3.

14. Table S1 caption should say "from" rather than "form".

We thank the reviewers for noting this error and have revised the text accordingly.

Reviewer #3: *The study by Boivin et al aims to define the cellular, molecular and circuit-level mechanisms of a form of adaptive modulation of nociceptive behavior in Drosophila. The authors use a combination of optogenetic and naturalistic behavior paradigms to convincingly demonstrate that chronic optogenetic stimulation drives lasting sensitization to nociceptive inputs. They further show that this sensitization involves heightened activation of nociceptive neurons to noxious inputs, requires octopamine receptor function in nociceptors, and identify neurons in the VNC that participate in the sensitization. Overall this is a well-designed study and results are generally clear and convincing (particularly behavioral evidence and calcium imaging demonstrating sensitization), though the physiological relevance of the phenomenology is not clear (point 1 below). The proposed mechanism of sensitization (increased OAMB expression) is not supported by the results provided, but this is not an essential element of the manuscript. Finally, the model for feed-forward control in the circuit is intriguing as this appears to represent an invertebrate model for central sensitization to noxious inputs, however this requires further substantiation (see points 3 and 4 below).*

1. The authors present compelling evidence that chronic optogenetic nociceptor stimulation drives nociceptive sensitization that persists for hours or days. However, the paradigms all rely on optogenetic stimulation for extended periods (24 h or more) to trigger the mechanism under study. The study would be substantially strengthened by identification of roles for this octopaminergic feed-forward mechanism in some physiologically relevant situation.

We thank the reviewers for highlighting this important point. To address it, we performed a new experiment examining the effects of acute nociceptor stimulation (5 seconds or 5 minutes repeated for one hour) on subsequent nociceptive behavior (Supplemental Figure S1A–C). These results show that acute stimulation is sufficient to modulate behavior, and that increasing the duration or chronicity of stimulation enhances the magnitude of the response.

Because the octopaminergic system is homologous to the mammalian stress system, this feed-forward mechanism may allow the nociceptive system to prime itself for future stressors, with repeated or prolonged activation producing progressively stronger behavioral responses. Our findings also reveal that the frequency of stimulation can determine whether the system undergoes habituation or sensitization, suggesting that the fundamental mechanisms regulating experience-dependent modulation of nociception are likely conserved across species.

2. The evidence that OAMB-R expression is responsive to nociceptor activation is not compelling. The studies with the transcriptional reporter suggest that OAMB-R levels might change in ddaC in response to activation, but no evidence is provided to evaluate whether modulating OAMB-R levels in ddaC is sufficient to induce this form of sensitization. Calcium imaging studies show that the enhanced calcium responses of ddaC to optogenetic stiumuls are attenuated by OAMB RNAi – do v'ada and At a minimum, the authors show use more direct assays to monitor OAMB-R RNA expression levels (in situs) to support their claims. Does acute OAMB expression (for example using the gene-switch system) sensitize nociceptors to noxious inputs?

We thank the reviewer for their insightful suggestion. To test whether overexpression of OAMB is sufficient to induce sensitization, we overexpressed OAMB in nociceptive sensory neurons. This experiment revealed that overexpression of one OAMB isoform (OAMB-AS) in these neurons was sufficient to robustly replicate the experience-dependent effect (Supplemental Figure S3G–I). The experiment is described in detail between lines 365 and 371.

3. The authors present syb-GRASP results that are suggestive of synaptic connectivity between C4da neurons and VUMs. Their calcium imaging results demonstratethat VUM responds to C4da activation and that prior C4da stimulation heightens VUM responses to VUM activity, however their studies as presented do not support the conclusions that prior activation of C4da neurons increases synaptic connectivity to VUM.

We thank the reviewer for this insightful comment. We agree that our original data do not provide sufficient evidence to conclude that prior activation of C4da neurons increases synaptic connectivity to VUM neurons. A more detailed analysis, such as quantifying individual synapses, is not currently possible because the syb-GRASP reagent is activity-dependent, complicating interpretation of fluorescence intensity, and available activity-independent GRASP reagents do not reliably localize to the relevant subcellular compartments.

Accordingly, we have revised the manuscript to remove this claim. We now emphasize that synaptic contacts between C4da neurons and VUMs exist and that our data support experience-dependent changes in functional connectivity. Specifically, lines 594 and 398 now refer to "functional connectivity" instead of "synaptic transmission," and lines 649 replace "synaptic and functional" with "functional connectivity.

4. The authors discuss a role for both pre- and post-synaptic changes in mediating sensitization. What is the evidence for post-synaptic changes in VUM neurons following prior stimulation of C4da neurons? In figure 6, the authors presesnt imaging studies showing that octopamine release onto C4da axons increases following prior octopaminergic stimulation. Do they see a similar effect to prior C4da stimulation? They should include this experiment as it provides a more compelling test of their model.

We thank the reviewer for this helpful suggestion. We performed the recommended experiment to directly test whether prior noxious stimulation increases octopaminergic input to C4da neurons. Despite a higher and more variable baseline compared to Figure 6H–I, the results are consistent with and support our proposed model. This experiment is shown in Figure 6J–K and described in detail between lines 612 and 620.

Minor

1. The details of the paradigm in 1F are not completely clear. Larvae are being stimulated in the indicated developmental window, but when is behavior being assayed? If they're all being assayed at the same time (120-144 h AEL?), the results don't necessarily support the conclusion that stimulation of older larvae generates more substantial potentiation if the effects decay after removal of the conditioning stimulus.

We thank the reviewer for their comment. We clarified the protocol in Figure1F and we are making to highlight this flaw. It must be noted that all larvae were tested at the same age (at 121 h AEL) and had for 1h of rest between the stimulation protocol and the test. That said, it would be difficult to test larvae at a stage other than the third instar, as previous studies suggest that C4da-evoked rolling is somewhat temporally restricted to that developmental stage.

We also agree that larvae stimulated at 0–24 h or 24–48 h exhibited weaker sensitization compared to those stimulated at 94–120 h, likely due to decay after removal of conditioning. Importantly, even 0–24 h ALE stimulation induces sensitization relative to naïve larvae, indicating that developmental nociceptive stimulation at early stages can also produce a sensitization phenotype. The text has been revised accordingly (line 183).

2. The study demonstrates that Octopamine feeding acutely sensitizes larvae to noxious stimuli. This result seems at odds with the model that OAMB induction plays an important role in sensitization.

We thank the reviewer for this comment. The newly performed experiments—OAMB overexpression in nociceptive neurons (new Figure S3G–I) and OAMB knockdown combined with octopamine feeding (new Figure 5G–H)—suggest that the sensitization mechanism relies on two distinct but related components: an increase in OAMB expression in C4da neurons and an increase in octopamine release from VUMs.

Notably, basal OAMB expression is sufficient to mediate the effects of octopamine feeding, and overexpressing OAMB in nociceptive neurons of naïve larvae partially mimics the effects of developmental nociceptor activation, likely by amplifying the detection of a normally occurring feedback loop (Supplemental Figure S3G–I)

3. Related to the octopamine feeding experiments. The authors should include cell-specific OAMB knockdown in C4da neurons to rule out the possible contribution of octopamine signaling through other da neurons to the observed responses. Does OAMB RNAi in C4da neurons block effects of octopamine feeding?

Thank you for the insightful suggestions. We have now tested OAMB RNAi in C4da neurons combined with octopamine feeding to determine whether the effects of octopamine feeding are mediated through OAMB in C4da neurons (Figure 5G–H). We found that knockdown of OAMB in C4da neurons blocks the effect of octopamine feeding, consistent with the conclusion that octopamine's effect requires OAMB in C4da neurons.

4. VUM stimulation drives only a modest increase in nociceptive behavioral output (~38% response for controls, ~50-52% response for tdc2/vum>chrimson; prior C4da stimulation or OA treatment yielded

~80% response in these assays. Why the modest sensitization to vum stimulation? The plots would be easier to follow if the naive and pre-stimulated conditions are shown together for each genotype

Thank you for the suggestion regarding the order of genotypes/conditions in Figure 6C. We have revised the order accordingly.

We would like to clarify the effect of VUM stimulation during development (Figure 6C). As the reviewers noted, when mechanical stimulation was used to evoke rolling behavior, 52% of animals responded under conditions activating VUMs, $tdc2^+$ neurons, or $tdc2^+$ with tshFLP (similar to VUMs), compared to 38% in control animals. These data are consistent with the conclusion that VUM stimulation during development evokes sensitization. Mechanical stimulation was used because optogenetic activation could not be applied to nociceptive neurons in these experiments, as Chrimson was already expressed in VUMs or $tdc2^+$ neurons.

In contrast, OA treatment or prior C4da stimulation produced an 80% rolling response when nociceptive sensory neurons were activated optogenetically (UAS-Chrimson with red light). Mechanical nociceptive stimulation evoked 52% or 65% rolling responses, similar to the VUM stimulation phenotype. The slightly smaller effect observed with VUM stimulation compared to C4da activation may reflect the additional cell-autonomous contribution of C4da neurons themselves, on top of feedback from tdc2 neurons.

5. The authors overstate the effects of octopaminergic silencing on the C4da sensitization. Their results (Fig. 4C) are consistent with tdc2-GAL4 neurons contributing to sensitization, but other neurons appear to contribute as well.

We thank the reviewers for their critical suggestions. We have added the following sentences at line 417-421.

"When $tdc2^+$ neuron activity was inhibited during C4da neuron activation, we observed a significant reduction, but not a complete elimination, of sensitization in developmentally stimulated larvae (tdc2 > –: 89.41 ± 6.54%, – > GtACR1 control: 86.0 ± 3.89%, tdc2 > GtACR1: 58.5 ± 4.5%) (Fig. 4C). This indicates that other neurons may also contribute to the sensitization phenotype."
