# Supplementary material for: A positive feedback loop between sensory and octopaminergic neurons underlies nociceptive plasticity in Drosophila larvae
Source: PLoS Genet. 2026 Apr 28;22(4):e1012122. doi: 10.1371/journal.pgen.1012122 (PMC13152156; doi:10.1371/journal.pgen.1012122)
Supplement: Attachment — Submitted filename: Responses_to Reviewer_Rev2.pdf [file pgen.1012122.s014.pdf]

To the Editors of PLOS Genetics:

We have revised the manuscript, titled "A positive feedback loop between sensory and octopaminergic neurons underlies nociceptive plasticity in *Drosophila* larvae", authored by Boivin et al., and additional comments provided by the reviewers, which have improved the clarity and rigor of our work. We have addressed reviewer questions.

Comments to the Authors:
Please note here if the review is uploaded as an attachment.

Reviewer #1: The authors have done a great job in addressing/clarifying my points of concern. I recommend acceptance of the manuscript.

Reviewer #3: The authors have been responsive to reviewers comments and substantially improved the manuscript. I still have some questions about the connection to OAMB. The authors show that OAMB RNAi attenuates calcium responses in ddaC (but not other class IV da neurons - why??) in response to optogenetic stimulation and provide additional studies showing that chronic overexpression of one isoform induces sensitization. However, the authors do not provide any direct evidence that the particular isoform is expressed in class IV da neurons, or that OAMB expression is responsive to activity (the GAL4 insertion is used as a proxy but it's not clear that this faithfully reports on the expression). Given the centrality of the receptor to the pathway, this seems like a point that should be addressed.

We thank the reviewer for raising this critical point.

1. Rationale for focusing on ddaC neurons

The main reason that we focus specifically on ddaC neurons was guided by both biological observations and technical considerations. Primarily, we noted that OAMB expression increased specifically within ddaC neurons following the stimulus—a localized response that was not consistently observed in other class IV da (C4da) neurons. This focus was further supported by the technical advantages of the ddaC model, which allows for more consistent and high-quality imaging of dendritic positions. Crucially, these cell-specific findings are reinforced by our additional axon terminal data (Figure S3E-F); while this data reflects the collective input from all C4da neurons, the resulting activity patterns remained fundamentally consistent with the observations recorded in ddaC dendrites.

2. Evidence for activity dependent OAMB isoform specificity

We agree that directly demonstrating the endogenous, isoform-specific expression of OAMB in C4da neurons would be ideal. Identifying endogenous, isoform-specific expression in C4da neurons remains technically demanding in our lab, because these cells comprise a very small population of peripheral sensory neurons, often exceeding the sensitivity limits of current isoform-resolved transcriptomic analysis or in situ mRNA hybridization. Nevertheless, analysis of available single-cell RNA-seq data (Tenedini et. al 2025, Boiko et. al. 2017) suggests that OAMB-AS is the primary isoform expressed in C4da neurons, while -KS3 isoform is not detected level.

In response to the reviewer's concern, we have revised the text to avoid over-interpreting these results. We now state that limitation of our data to conclude that OAMB-AS is critical for the sensitization and further study is necessary in discussion (line 670-678). We also revised the OAMB-Trojan GAL4 data in terms of reporter expression rather than OAMB expression, ensuring a more cautious interpretation of genetic data.

While direct isoform-specific expression data would strengthen the study, we believe the combination of cell-specific knockdown, isoform-specific gain-of-function experiments, and public available single-cell RNA-seq data provides strong functional evidence that OAMB operates within C4da neurons to mediate the observed sensitization. We have updated the manuscript to clearly delineate the limits of these conclusions and to suggest avenues for future molecular characterization.
